# Secretory Proteomic Responses of Endometrial Epithelial Cells to Trophoblast-Derived Extracellular Vesicles

**DOI:** 10.3390/ijms241511924

**Published:** 2023-07-25

**Authors:** Subhashini Muhandiram, Keerthie Dissanayake, Toomos Orro, Kasun Godakumara, Suranga Kodithuwakku, Alireza Fazeli

**Affiliations:** 1 Institute of Veterinary Medicine and Animal Sciences, Estonian University of Life Sciences, Kreutzwaldi 62, 51006 Tartu, Estonia; subhashini@emu.ee (S.M.); keerthiedissanayake@yahoo.com (K.D.); toomas.orro@emu.ee (T.O.); kasun.godakumara@emu.ee (K.G.); suranga.kodithuwakku@emu.ee (S.K.); 2 Department of Pathophysiology, Institute of Biomedicine and Translational Medicine, University of Tartu, Ravila St. 14B, 50411 Tartu, Estonia; 3 Department of Anatomy, Faculty of Medicine, University of Peradeniya, Kandy 20400, Sri Lanka; 4 Department of Animal Science, Faculty of Agriculture, University of Peradeniya, Kandy 20400, Sri Lanka; 5 Academic Unit of Reproductive and Developmental Medicine, Department of Oncology and Metabolism, Medical School, University of Sheffield, Sheffield S10 2TN, UK

**Keywords:** embryo, endometrium, extracellular vesicles, secretome, proteomics, epithelial cells, trophoblast cells

## Abstract

Synchronized crosstalk between the embryo and endometrium during the periconception period is integral to pregnancy establishment. Increasing evidence suggests that the exchange of extracellular vesicles (EVs) of both embryonic and endometrial origin is a critical component of embryo–maternal communication during peri-implantation. Here, we investigated whether embryonic signals in the form of EVs can modulate the endometrial epithelial cell secretome. Receptive endometrial analog RL95-2 cells were supplemented with trophoblast analog JAr cell-derived EVs, and the secretory protein changes occurring in the RL95-2 cells were analyzed using mass spectrometry. EVs of non-trophoblastic origin (HEK 293 cells) were used as the control EV source to supplement endometrial cells. Trophoblast cell-derived EVs enriched endometrial epithelial cell secretions with proteins that support embryo development, attachment, or implantation, whereas control EVs were unable to induce the same effect. The present study suggests that embryonic signals in the form of EVs may prime receptive endometrial epithelial cells to enrich their secretory proteome with critical proteomic molecules with functional importance for periconception milieu formation.

## 1. Introduction

Advances in assisted reproduction technologies (ARTs) and infertility treatments have shown limited progress in improving human fertility, and embryo implantation failure remains a major cause of pregnancy failure in humans [1,2]. Synchronized crosstalk between the embryo and receptive endometrium during peri-implantation is required for a successful pregnancy. Embryo implantation is a highly regulated process wherein the competent blastocyst interacts with the receptive endometrium (apposition), attaches to the endometrium (attachment), and then invades the endometrium and maternal circulation to form the placenta (invasion) [3]. Numerous endocrine, paracrine, and autocrine modulators govern the initial communication between the outer layer of the embryo, known as the trophectoderm, and the endometrium when in close proximity [4]. During this time, the embryo undergoes the intrinsic molecular reprogramming of cellular growth and differentiation, whereas the endometrium undergoes temporal and spatial differentiation to achieve endometrial receptivity [5]. Impairment of reciprocal molecular communication between the embryo and the endometrium during this time interrupts uterine receptivity and impedes embryo development to implantation competency, which subsequently may lead to implantation failure or embryo rejection even after breaching in to the decidualized stroma [6].

A variety of cytokines, growth factors, transcription factors, extracellular matrix-related enzymes or inhibitors, and adhesion molecules of both embryonic and uterine origin are involved in the structural and functional remodeling of endometrial cells for embryo reception [7]. However, the exact molecular and hormonal pathways involved remain elusive. Extracellular vesicles (EVs) are a heterogeneous group of secretory messengers that mediate the novel mechanism of intercellular communication by delivering their bioactive cargo, including proteins, RNA, lipids, and DNA, to adjacent and distant recipient cells. The production of EVs from pre-implantation embryos in vitro has been reported in several species, including humans [8,9,10]. EVs from embryonic or trophoblast cells are then uptaken in an autocrine [11] and/or paracrine manner [8]. Embryo-derived EVs have the ability to alter the gene expression profile of oviductal epithelial cells, and this effect is dependent on embryo quality [9]. These findings suggest that EVs secreted by the embryo may carry a specific message to the maternal tract, providing information about the presence and quality of the embryo. Increasing evidence suggests that embryonic-derived EVs may also deliver their bioactive cargo to the endometrium, potentially impacting the phenotype and activity of endometrial cells in the early phases of embryo implantation [8,12,13,14]. In our previous reports, we demonstrated that trophoblast cell-derived EVs can specifically reprogram the endometrial epithelial cell transcriptome to support early embryo–maternal communication. Our results suggested that the gene expression in endometrial epithelial cells could be affected by the specific miRNA cargo content of trophoblastic EVs- [13]. Accumulating evidence also suggests that trophectoderm-derived EVs can modulate the proteomic repertoire of endometrial epithelial cells to acquire the receptive phenotype [14]. Trophoblast-derived EVs not only have a specific miRNA profile but also possess a distinctive protein cargo [14,15]. However, the exact mechanisms of how trophoblast EV miRNA or protein cargo induce favorable changes in endometrial cells are not completely understood. Recent studies have shown that human blastocysts are capable of taking up EVs from endometrial epithelial cells, carrying miRNA cargo with functional importance for embryo development and implantation [16,17,18]. Another study showed that EVs derived from endometrial cells play a crucial role in modulating the trophectoderm cell secretome, thereby promoting embryo implantation [19]. According to the literature, cargo in EVs derived from endometrial cells carry messages about endometrial receptivity status. Specifically, EVs obtained from receptive endometrial cells were capable of enhancing embryo potency, while those derived from non-receptive endometrial cells did not exert the same effect [18]. This evidence depicts the potential role of EV exchange as an emerging bidirectional mode of communication at the embryo–maternal interface during the periconception period. However, how trophoblast EVs alter the endometrial epithelial cell secretome and what properties of EVs can be attributed to such changes are understudied facets.

The endometrial microenvironment consists of a mixture of secretions from both the embryo and endometrium that change consistently during the pre-conception period. These changes support the growth and development of blastocysts to establish a pregnancy [20,21,22]. The role of estrogen and progesterone signaling in the endometrium in secreting a supporting medium-targeting embryo has been demonstrated in multiple studies [23,24]. A study by Haart et al. reported that endometrial-cell-derived EVs released during the receptive period can contain proteins that support embryo implantation [22]. Notably, EVs and the secretome of hormone-primed receptive endometrium have proven capable of inducing cellular adhesion and outgrowth in trophectoderm cell spheroids, increasing implantation rates [14,25,26]. Hence, various signaling molecules have the potential to modulate the secretome and the EV cargo composition of endometrial epithelial cells. This modulation is crucial in preparing an embryo for successful implantation.

Recent studies have clearly demonstrated the significance of estrogen and progesterone hormones in controlling the secretome and EV cargo of endometrial epithelial cells. However, whether the EVs from the embryo, a novel form of signaling, can prime endometrial epithelial cell secretions and embryo implantation remains unknown. In the present study, we used an in vitro cell culture model consisting of JAr cells (to mimic pre-implantation trophoblast cells) and RL95-2 cells (to mimic receptive endometrial epithelial cells) to simulate the embryo–maternal interface during the periconception period [12,13]. Using this in vitro model, we demonstrated that trophoblast-derived EVs have the ability to alter the secretory proteome of endometrial cells to ensure that they contain key molecules that may facilitate embryo development and implantation. Moreover, the identification of the secretory protein responses of endometrial cells to embryonic signals will enable a better understanding of the exact signaling pathways and networks that orchestrate the implantation process.

## 2. Results

### 2.1. Trophoblast Cell-Derived EVs Induce Specific Secretory Protein Response in Endometrial Epithelial Cells Compared to Non-Trophoblast Cell-Derived EVs

**Figure 1:**
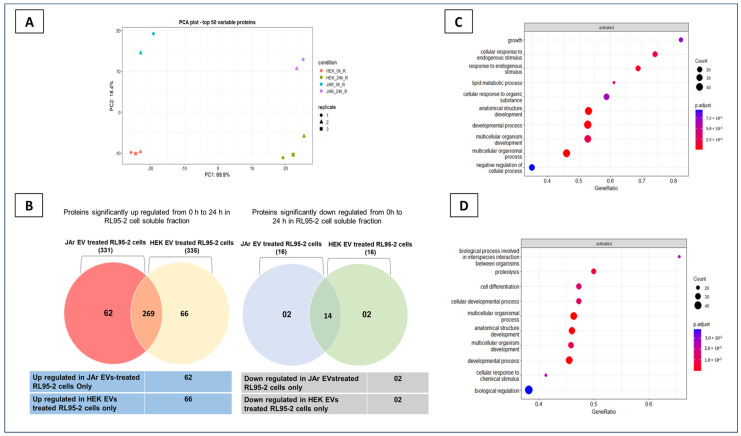
The secretory proteomic profiles of the RL952 cells responding to JAr EVs were distinguishable from RL95-2 cells treated with HEK EVs after 24 h of treatment. (**A**) Variation in the protein expression profiles of secretory components of RL 95-2 cells after treatment with JAr and HEK EVs at 0 h and 24 h. Two principal components of the first 50 variable proteins are shown. JAr EV-treated RL95-2 secretory protein profile at 0 h; JAR_0h_R, JAr EV-treated RL 95-2 secretory protein profile at 24 h; JAR_24h_R, HEK EV-treated RL 95-2 secretory protein profile at 0 h; HEK_0h_R, HEK EV-treated RL 95-2 secretory protein profile at 24 h; HEK_24h_R. (**B**) Venn diagram of proteins identified in RL95-2 cell-conditioned media significantly changed from 0 h to 24 h in response to JAr and HEK cell-derived EVs (proteins with fold change log2 > 1, FDR < 0.05 were considered upregulated proteins from 0 h to 24 h, and proteins with fold change log2 < −1, FDR < 0.05 were considered downregulated proteins). Up- and downregulated proteins from 0 h to 24 h in JAr and HEK EV-treated groups were determined separately, and common and unique protein changes in each treatment group are shown in the Venn diagram. (**C**) Gene set enrichment analysis of all proteins significantly altered in the JAr EV-treated RL95-2 cell secretome from 0 h to 24 h. (**D**) Gene set enrichment analysis of all the proteins significantly altered in the HEK 293 EV-treated RL95-2 cell secretome from 0 h to 24 h.

JAr and HEK 293 cell-derived EVs were isolated, and RL952 cells were treated with each EV type at a concentration of 1 × 10^9^ nanoparticles/mL [12,13]. Secretory proteomic alterations in RL95-2 cells in response to JAr and HEK EVs were identified. The proteomic profiles of cell culture supernatants of RL95-2 cells at 0 h and 24 h after treatment with EVs were analyzed to identify differential enrichment of proteins. In total, 1023 proteins were identified (including 142 explicitly identified protein isoforms and 22 uncharacterized proteins) in RL95-2 cell culture-conditioned media. The total number of proteins identified in the 24 h post-treatment samples was much higher than that in the 0 h samples (Figure S3). Therefore, it was apparent that, in the presence of EVs, the expression of multiple proteins in the RL95-2 cell secretome changed between 0 h and 24 h. The protein expression profiles of the top 50 variable proteins in principal component analysis (PCA) are shown in Figure 1A. One replicate of the JAr EV-treated RL95-2 cells, which was identified as an outlier in the PCA, was excluded from the analysis (Figure S3). The secretory protein changes occurring in the JAr EV-treated RL95-2 cells were distinguishable from the HEK EV-treated RL95-2 cells after 24 h of incubation (Figure 1A,B). The fold change regarding proteins identified only in the JAr EV-treated RL95-2 cell secretome was plotted against the average protein abundance of the proteins across all samples (Figure S4). Proteins such as PRDX2, MIF, LDHA, PGK1, etc., were highly abundant in the cell culture-conditioned media and changed in the JAr EV-treated RL95-2 secretome. Interestingly, these proteins are known to have roles in the process of embryo implantation [14,27,28,29].

Gene set enrichment analysis of all of the proteins that were significantly altered (from 0 h to 24 h) in the JAr EV-treated group revealed “growth” and “cellular response to endogenous stimulus” as the most enriched pathways (Figure 1C). On the other hand, in the HEK EVs-treated group, “biological process involved in interspecies interaction between organisms” and “proteolysis” were identified as the most enriched pathways (Figure 1D). Overall, JAr EV treatment induced specific secretory protein changes in the RL95-2 cell secretome that were clearly distinguishable from the control EV (HEK EVs)-treated RL95-2 cell secretome.

### 2.2. Specific Secretory Protein Changes in RL95-2 Cells in Response to JAr Cell-Derived EVs Reveal Potential Players of Embryo Implantation

The specific secretory protein alterations in RL95-2 cells attributed to JAr and HEK EVs treatment were sorted, and functional annotation and KEGG pathway analysis were performed using the Database for Annotation, Visualization, and Integrated Discovery (DAVID 6.8) online tools (version DAVID 6.8) (Figure 1B). JAr EVs induced specific secretory proteins in RL95-2 cells, showing enrichment in pathways such as “glutathione metabolism” (a key antioxidant response pathway), “amino acid metabolism”, “glycolysis”, “tight junctions”, and “protein processing in endoplasmic reticulum” (*p* ≤ 0.05). These pathways have been previously implicated in the processes of embryo development and implantation [14,30,31] (Table 1). Proteins expressed specifically in RL95-2 cells in response to HEK EVs revealed no such significant pathways in the context of embryo implantation (Supplementary Table S1).

The proteins specifically altered in the JAr EV-treated group from 0 h to 24 h were subjected to functional annotation and GO enrichment pathway analysis. In the results, proteins involved in multiple cellular metabolic pathways such as “cellular responses to oxidative stress”, “redox homeostasis”, and “glutathione metabolism” were significantly enriched (Supplementary Figure S3A). The major proteins related to these GO enrichment pathways were GSR, PRDX2, PRDX6, SOD1, PRDX1, and GSTP1, and most of these antioxidant enzymes have been previously reported to change inside endometrial epithelial cells in response to trophectoderm-derived EVs [14]. Our GO enrichment analysis of the proteins specifically altered in the HEK EV-treated group from 0 h to 24 h varied completely from the JAr EV-treated group and did not indicate any significant pathways related to embryo implantation (Figure S5).

Manual top-down data mining of differentially expressed proteins enabled the identification of 24 more proteins that are often involved in the process of embryo implantation, endometrial receptivity, or embryo development (Table 2).

**Figure 2:**
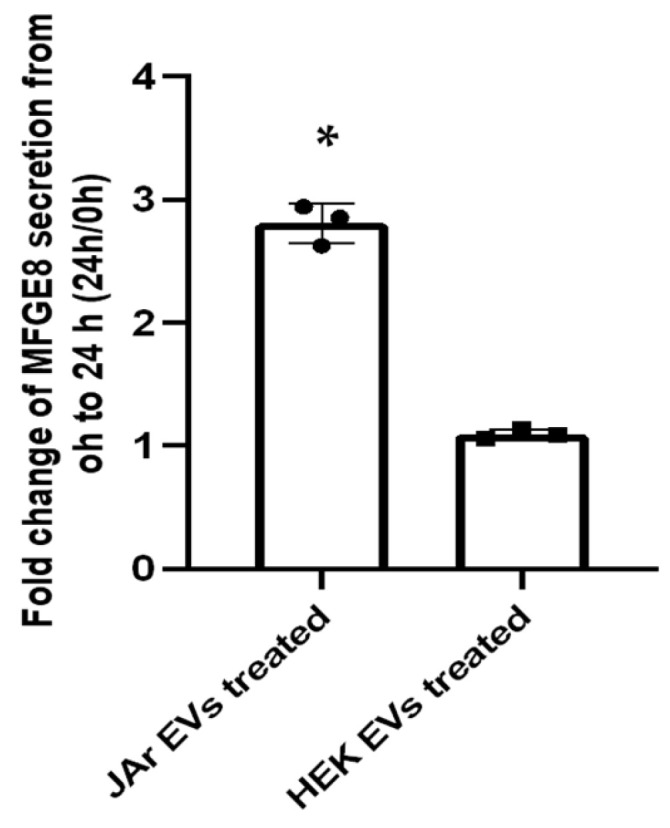
Verification of mass spectrometry data for the MFGE8 protein. JAr EV-treated RL95-2 cells significantly increased the secretion of MFGE8 proteins between 0 h and 24 h, while HEK EV-treated RL95-2 cells were unable to induce the same effect. The asterisk indicates *p* < 0.05.

Orthogonal validation of the mass spectrometry data was performed on the MFGE8 protein using ELISA (Figure 2).

Here, the data indicate that trophoblast-derived EVs can enrich the endometrial cell secretory proteome with known players of embryo implantation.

## 3. Discussion

Successful embryo implantation is attributed to the synchronized development and differentiation of both the endometrium and the embryo [33]. The endometrium becomes receptive to the embryo only during the short window of implantation, characterized by the optimal cellular and molecular transformation of the endometrium and reciprocal interactions between the embryo and endometrium through a range of signaling molecules [32]. Previously, multiple studies have shown that trophoblast or trophectoderm-derived EVs can signal endometrial epithelial cells to acquire a receptive phenotype [12,13,14]. EVs generated by embryonic [13,14] or endometrial cells [19,22] have distinct protein [14,22] and RNA [13] cargos which can be internalized by the endometrium or embryo [8,11]. Embryonic signals in the form of EVs have been shown to alter the transcriptomic [13] and proteomic [14] landscapes of endometrial cells. These changes potentially contribute to critical components of embryo–maternal communication during peri-implantation. Nevertheless, whether embryonic cell-derived EVs can modulate the endometrial cell secretory proteome is not yet known.

In the current study, we demonstrated that trophoblast-derived EVs can prime endometrial epithelial cell secretions to ensure they contain the critical proteins involved in the process of embryo implantation. Importantly, EVs derived from non-trophoblastic cells (HEK 293 cells) did not induce the same effect in the endometrial cells, which is in line with our previous report on the effects of trophoblast-derived EVs on the endometrial cell transcriptome [13]. The proteomic profile of JAr EV-treated RL95-2 cell culture supernatant (JAR_0h_R) was distinguishable from HEK 293 EV-treated cell culture supernatant (HEK_0h_R) in the PCA plot, even at 0 h (Figure 1A). This suggests inherent differences in the molecular cargos of JAr and HEK EVs. However, we assume that, after 24 h, the supplemented EVs are either internalized or degraded, leading to changes in endometrial epithelial cell secretome. Each EV type can have a unique molecular signature depending on its cell of origin, which can mediate specific biological functions in the recipient cells [70,71,72]. To account for the unique molecular signature of JAr and HEK EVs, we used the 0 h samples of JAr and HEK EVs containing cell culture supernatants as the baseline samples. However, the exact mechanism by which the protein signatures of EVs contribute to specific biological functions in recipient cells is not fully understood. The enrichment pattern of our proteomic results was further validated by a significantly high level of MFGE8 protein secretion in the JAr EV-treated RL95-2 cells secretome compared to the control EV-treated RL95-2 cell secretome.

Bioinformatics analysis revealed that glutathione metabolism (a key antioxidant response pathway) [30,69,73], amino acid metabolism [74,75], glycolysis [69,76], tight junctions [14,77,78], and protein processing in the endoplasmic reticulum [79,80] were highly upregulated in the endometrial epithelial cells in response to the trophoblast-derived EVs. Interestingly, all these proteins play critical roles in embryo implantation. Moreover, the major subset of proteins uniquely identified in the JAr EV-treated RL95-2 cell secretome consisted of antioxidant enzymes, including PRDX1/2/6, SOD1, GSR, and GST. The optimum balance of reactive oxygen species (ROS) in the uterine microenvironment is vital for early embryonic development and the regulation of innate and acquired immunity in embryonic cells [59,81]. Glutathione is a major cellular antioxidant that regulates ROS production. Glutathione and other antioxidative enzymes such as superoxide dismutase (SODs) can remove ROS and create a favorable microenvironment for embryo development [82,83]. Antioxidant activity in the vicinity of the embryo can improve in vitro fertilization (IVF) efficiency and subsequent embryo development [73]. SOD activity is elevated during the window of implantation and early pregnancy in the endometrium, suggesting its potential role in endometrial receptivity and embryo implantation [57]. Furthermore, JAr EV treatment caused secretory protein changes related to glycolysis or gluconeogenesis in RL95-2 cells, which are critical for maintaining antioxidant defense systems [84]. The proteins found in this study—GPI, LDHA, and PGK1—are known to be abundant in avian uterine fluid [85]. The same proteins are upregulated in uterine fluid during the maternal recognition of pregnancy in mares [60]. Furthermore, proteins related to cellular tight junction formation and the maintenance signaling pathway were increased in endometrial epithelial cells upon exposure to trophoblastic EVs. The same observation was reported recently, where an increase in transepithelial resistance (TER) was observed in Ishikawa cells after treatment with trophectoderm-derived EVs. Notably, all these pathways and most of the protein changes were reportedly highly upregulated in the intracellular proteome of the Ishikawa cells primed with trophectoderm-derived EVs in the same study, further validating the results of the present study [14].

The transformation of the endometrium, which ensures they acquire receptive features, occurs in the mid-secretory phase (day 20–24) of the human menstrual cycle [86]. Major changes in the endometrium during this period have been linked to extracellular matrix remodeling [87,88] and epithelial cell membrane or cytoplasmic rearrangement (to acquire apical cell polarity) [89,90]. Once the embryo reaches the receptive endometrium, it undergoes cellular growth and differentiation to facilitate implantation [91]. Thus, the peri-conception and implantation microenvironment is highly enriched in adhesion molecules, growth factors, and immune-related proteins such as cytokines [92]. Manual data mining of our dataset also revealed additional protein markers involved in embryo implantation, endometrial receptivity, and embryo development in the endometrial secretome after exposure to trophoblastic EVs. Through proteomic profiling, we identified that trophoblast-derived EVs can prime endometrial cells to secrete the critical proteomic factors of embryo development (PCNA [34], GSR [35,36], MAT2A [38], UBE2L3 [44], PRDX2 [14,27], PRDX6 [14,52], STIP1 [56], SOD1 [14,57], GSTP1 [14,60], and MYH9 [62]), endometrial differentiation (COL5A2 [33], XPO [39], LGALS3 [14,42,43], PGK1 [53]), and embryo attachment or implantation (MFGE8 [14], CCT8 [46], LDHA [14,31]). The factors that change in the embryonic vicinity can potentially alter the epigenome of the peri-implanting embryo, subsequently affecting embryonic gene expression, metabolism, and developmental capacity [37]. The changes in proteins associated with epigenetic modifications such as DNA methylation (e.g., MAT2A) in the JAr EV-treated group suggest that these changes in the feto–maternal interface can have long-term effects on the future development of the fetus, and this can even continue into adult life and affect future generations.

**Figure 3:**
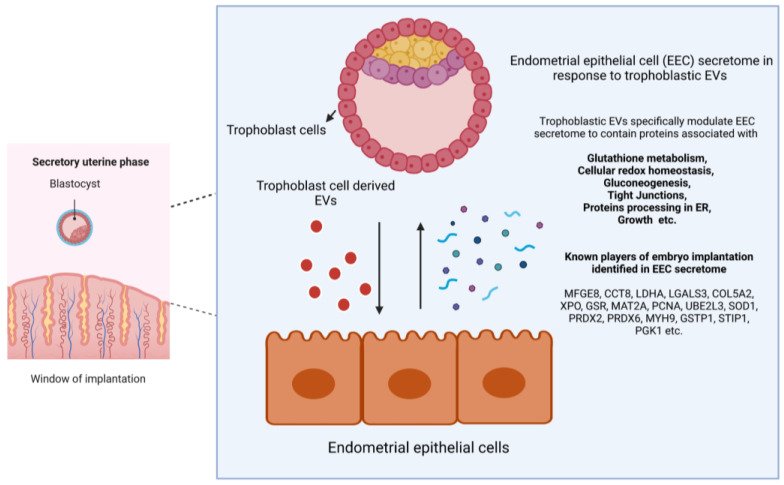
Trophoblast cell-derived EVs can modulate the endometrial epithelial cell secretory protein repertoire to facilitate embryo implantation.

Hence, it can be postulated that the secretory protein changes that occur in endometrial epithelial cells in response to trophoblast cells can modulate the immediate and long-term effects that function both in an autocrine and/or paracrine manner in the embryo–maternal interface during the peri-implantation period (Figure 3).

Major protein changes were associated with glutathione metabolism, cellular responses to oxidative stress, cellular redox homeostasis, gluconeogenesis, tight junctions, and protein processing in the endoplasmic reticulum (ER). All these pathways may have critical roles in the embryo–maternal interface during pre-implantation. In addition to antioxidant activity-related proteins, several other known proteins that play a role in endometrial receptivity, embryo implantation, and early embryo development were also identified.

The in vitro model we used consisted of JAr and RL95-2 cells and is a well-established cell culture model that has been frequently used in our laboratory for many other studies to simulate embryo–endometrial interactions [12,13,93,94]. However, there is a need in the field for more advanced models to simulate the embryo–maternal interface during the peri-conception period. There are serious ethical concerns in utilizing real human embryos for experimentation. Although fixed human tissues can be obtained and provide information about the cellular location of molecules, they are not suitable for functional studies. Hence, in vitro models utilizing cell lines have facilitated the study of embryo–maternal interactions on a functional level. In this study, JAr cells derived from first-trimester choriocarcinoma cells (which possess trophoblastic cell characteristics) were used as a renewable source of cells for EV isolation. This approach was chosen due to the difficulty in obtaining large amounts of freshly isolated trophoblast cells for the continuous production of EVs. We used RL95-2 cells to simulate a receptive endometrial cell line that facilitates the initial communication between the embryo and endometrium during peri-implantation. RL95-2 cells are generally used as a model of receptive endometrial epithelial cells. While there are drawbacks in in vitro systems, primarily due to the fact that these cells originate in cancer, many other models of human embryo implantation also have their own limitations. For example, studying embryo implantation using mouse models is deemed suitable for simulating the in utero environment; however, it is limited by the fact that the embryo implantation process is species-specific. Even differentiated embryonic stem cells have been shown to exhibit different adhesion capacities based on the epithelial cell lines used in the receptive end (Ishikawa or primary cells) [93].

It is likely that the endometrial epithelial cell secretome changes reported in this study are due to either EV uptake or EV signaling by binding to the endometrial epithelial cell membrane at ligand–receptor interaction-level (upstream). The effects of EVs can also be driven by cargo release in the epithelial cell membrane or inside the cell by binding to the ER. It is known that miRNAs secreted by blastocysts modulate the different cellular processes related to implantation [95]. Previously, we showed that trophoblast-derived miRNAs can be packaged in EVs and partly regulate gene expression in endometrial epithelial cells using the same in vitro model [13]. Emerging evidence also suggests that trophoblast-derived EVs can carry a unique protein cargo [14]. Hence, secretory protein changes in endometrial epithelial cells can be attributed to the unique molecular cargo content of trophoblastic EVs. Nevertheless, further studies are required to dissect different cargo molecules in trophoblastic EVs. Furthermore, comprehensive investigations are required to detect and understand the EV signaling and cargo release mechanisms inside endometrial epithelial cells. These studies will pave the way to better understand how specific EV messages are decoded by selected recipient cells to induce desired molecular changes.

## 4. Materials and Methods

### 4.1. Cell Culture

The human endometrial adenocarcinoma cell line, RL95-2, was obtained from American Type culture Collection (ATCC CRL-1671, Teddington, UK). The human choriocarcinoma cell line, JAr (HTB-144™, Teddington, UK), and human embryonic kidney cells, HEK 293 (ATCC^®^, CRL-3216™, Teddington, UK), were also purchased from ATCC. RL95-2 cells were routinely maintained in Dulbecco’s Modified Eagles medium F12 (DMEM 12-604F, Lonza, Verviers, Belgium) supplemented with 10% Fetal Bovine Serum (FBS) (Gibco™, 10500064), 1% penicillin streptomycin (P/S) (Gibco™, 15140122, Bleiswijk, The Netherlands), and 5 μg/mL insulin (human recombinant insulin, Gibco™, Invitrogen, Denmark) in 5% CO_2_ at 37 °C. The JAr cells were grown in T75 flasks containing RPMI 1640 medium (Gibco™, Inchinnan, UK) supplemented with 10% FBS, 1% P/S, and 1% L-glutamine in 5% CO_2_ at 37 °C. The HEK 293 T cell line was used as a source of control EVs. Briefly, the HEK 293 cells were grown in T75 flasks in DMEM F12 medium supplemented with 10% FBS, 1% P/S, and 1% L-glutamine in 5% CO_2_ at 37 °C. The media were changed every second day until the cells reached 80% confluency.

### 4.2. Preparation of EV-Depleted Media

EV-depleted medium was prepared as previously described [12,13,96]. In summary, FBS was filtered using Amicon^®^ Ultra-15 centrifugal filters with a 100 kDa cut-off (Merk KGaA, Darmstadt, Germany) at 3000× *g* for 55 min. The EV-depleted FBS was 90% depleted of nanoparticles and used at a 10% concentration to supplement all complete culture media specific to each cell type mentioned above.

### 4.3. EV Isolation and Characterization

At 80% confluency, the JAr and HEK 293 cell culture-conditioned media were replaced with EV-depleted culture media. After 24 h, the cell culture supernatants were collected and centrifuged at 400× *g* for 10 min to remove contaminating cells. The resulting supernatant was centrifuged again at 4000× *g* for 10 min, followed by centrifugation at 10,000× *g* for 10 min to remove cellular debris. The collected media were concentrated to a final volume of 500 µL using Amicon^®^ Ultra-15 centrifugal filters with a 10 kDa cut-off. Next, the EVs were purified using size-exclusion chromatography (SEC). A gel filtration medium consisting of 4–6% agarose matrix was used in columns that were 15 cm in length to separate the EV fractions from contaminating proteins. Fractions 7–10 were collected (each fraction was 500 µL in volume) and concentrated again to a total volume of 500 µL using an Amicon^®^ Ultra centrifugal filter device with a 10 kDa cut-off. Isolation and characterization of the isolated EVs was carried out using methods described in detail elsewhere and according to ISEV 2018 guidelines [12,13,97]. In summary, both the size and concentration of nanoparticles in the EV fractions were measured using Nano Particle Tracking Analyser (Particle Metrix GmbH, Inning am Ammersee, Germany). Transmission electron microscopy was used for the physical characterization of the EVs. The enrichment of EV surface protein markers CD 9, CD 63, and CD 81 was confirmed via western blotting. The methods used for JAr and HEK 293 cell-derived EV characterization have been fully described in our previous publications [12,13,97]. EVs derived from JAr cells and HEK 293 cells were referred to as JAr EVs and HEK EVs, respectively. JAr and HEK 293 cell-derived EVs with a high purity were obtained using size-exclusion chromatography with a nanoparticle distribution within a similar size range. The original nanoparticle concentrations and size profiles of JAr and HEK EVs are shown in Figure S1.

### 4.4. Collection of Cell Culture Supernatants for Secretory Proteome Analysis and Enzyme-Linked Immunosorbent Assay

Cell culture supernatants (1 mL of media) were collected from RL95-2 cells seeded in 12-well plates (seeding density of 1 × 10^6^ cells per well) and centrifuged at 400× *g* for 10 min to remove any contaminating cells; this was followed by centrifugation at 4000× *g* for 10 min and 10,000× *g* for 10 min to remove other cellular debris and apoptotic bodies.

### 4.5. Protein Quantification and Identification with Liquid Chromatography and Tandem Mass Spectroscopy (LC-MS/MS)

The total protein precipitation of cell culture supernatant samples was performed overnight with trichloroacetic acid deoxycholate (TCA-DOC). Approximate protein quantities were estimated based on the size of the pellets. The pellets were then solubilized in 7 M urea, 2 M thiourea, 100 mM ammonium bicarbonate (ABC), and 20 mM methylamine buffer. Protein reduction was performed via incubation for 1 h at room temperature using 5 mM dithiothreitol (DTT). Protein alkylation was performed via incubation for 1 h at room temperature in the dark using 10 mM chloroacetamide. Next, protease LysC (Wako, Monza, Italy) was added to an enzyme/substrate ratio (E:S) of 1:50, and the samples were incubated for 1 h at room temperature. The samples were then diluted five times with 100 mM ABC, and trypsin (Sigma Aldrich, St. Louis, MO, USA) was added at a 1:50 E:S ratio and incubated overnight at room temperature. After digestion, the samples were acidified with trifluoroacetic acid (TFA) at a concentration of 1%; subsequently, the samples were desalted on in-house made C18 StageTips. Then, the samples were reconstituted in 0.5% TFA, and peptide concentrations were determined using the Pierce colorimetric peptide assay (Thermo Fisher Scientific, Waltham, MA, USA). Next, 1 µg of peptide was injected into an Easy-nLC 1000 system^®^ (Thermo Scientific) and eluted at 250 nL/min from the trap to a 75 µm ID × 50 cm emitter column (New Objective) packed with C18 material (3 µm, 300 Å particles, Dr Maisch). The separation gradient was 2–35% B for 60 min and 40–100% B for 5 min (A: 0.1% formic acid (FA), B: 80% ACN + 0.1% FA). The eluted peptides were sprayed into a Q Exactive Plus^®^ (Thermo Fisher Scientific) quadrupole-orbitrap mass spectrometer (MS) using nano-electrospray ionization at 2.4 kV (applied through liquid junction). The MS was operated with a top 5 data-dependent acquisition strategy. Briefly, one 350–1400 *m*/*z* MS scan at a resolution setting of R = 70,000 at 200 *m*/*z* was followed by high-energy collisional dissociation fragmentation (normalized collision energy of 26) of the five most intense ions (z: +2 to +6) at R = 17,500. The MS and MS/MS ion target values were 3 × 10^6^ and 5 × 10^4^ (with an injection time of 50 ms). Dynamic exclusion was limited to 40 s. Mass spectrometric raw files were processed using the MaxQuant software package (versions 1.6.15.0 and 2.0.3.0). Methionine oxidation, asparagine and glutamine deamidation, and protein *N*-terminal acetylation were set as variable modifications, whereas cysteine carbamidomethylation was defined as a fixed modification. Label-free protein quantification (LFQ) was enabled using LFQ mode, and the protein minimum ratio count was set to 1. A search was performed against *Homo sapiens* and *Bos taurus* reference proteomes using the tryptic digestion rule. The peptide–spectrum match and protein false discovery rate (FDR) were kept below 1% using a target-decoy approach. All other parameters were set to default values. Mass spectrometry data are available in ProteomeXchange Consortium via the PRIDE with the dataset identifier PXD040311.

### 4.6. Differential Protein Expression and Bioinformatics Analysis

Differential protein expression between the treated and control groups was determined using R software version 4.2.1. using the DEP package (used for the analysis of mass spectrometry proteomics data for differential protein expression or differential enrichment). The DEP package for label-free proteomic data is extensively described in https://bioconductor.org/packages/devel/bioc/vignettes/DEP/inst/doc/DEP.html (accessed on 23 November 2022) [97]. Potential contaminating proteins and those originating from reverse sequencing were filtered out. Proteins with ≥2 valid values in at least one sample group were used for the analysis. Data were normalized using Variance Stabilizing Normalization (VSN). Missing values were imputed under low-intensity assumption, and statistical comparisons among the samples were performed using protein-wise linear models combined with empirical Bayesian statistics. Multiple testing was corrected using the Benjamini–Hochberg FDR (BH-FDR) [98]. Proteins were considered significantly differentially expressed if the fold change in protein expression was (FC) log2 > 1 or log2 < −1 and FDR < 0.05 between the 0 h and 24 h samples. Lists of the differentially expressed proteins between 0 h and 24 h samples were made for both the JAr and HEK EV-treated groups and then compared. The average protein abundances of the proteins across all samples were calculated, and the first and third quartiles of the data distribution were used to identify the most and least abundant protein groups.

Functional annotation and KEGG pathway analysis were performed using the DAVID Bioinformatics platform (https://david.ncifcrf.gov/, accessed on 23 November 2022). Functional annotation, GO pathway analysis, and gene set enrichment analysis were performed using the clusterProfiler package in R (https://bioconductor.org/packages/release/bioc/html/clusterProfiler.html, accessed on 23 November 2022). All relevant differentially enriched gene IDs were submitted to the DAVID Bioinformatics platform for KEGG pathway enrichment analysis. A complete protein list with log2 fold change and FDR was submitted to clusterProfiler to perform pathway overrepresentation analysis and visualization.

### 4.7. Verification of Proteomic Data Using Enzyme-Linked Immunosorbent Assay (ELISA)

To validate the mass spectrometry-based protein expression data, Milk fat globule-EGF factor 8 protein (MFGE-8) concentration was measured in the EV-treated cell culture supernatants using a commercially available ELISA kit (Human MFG-E8 Quantikine ELISA Kit, R&D systems) according to the manufacturer’s instructions. Briefly, 150 µL of the cell culture supernatant from each sample was diluted twice with the sample diluent provided with the kit. Then, the assay diluent was added to the precoated 96-well plate, followed by 100 µL of the sample, standard, or control. After two hours of incubation, the wells were washed and incubated with the MFGE-8 conjugate for 2 h. Next, the wells were washed, filled with 200 µL of substrate solution, and incubated for 30 min. Finally, stop solution was added, and the optical density of each well was measured using a microplate reader set to 450 nm (Multiskan FC microplate photometer, Life Technologies, Chongqing, China). The MFGE8 protein concentration changes in the JAr and HEK EV-treated groups from 0 h to 24 h were shown as mean ± SD (fold change expression). Statistical significance was determined using Student’s *t*-test, and results were considered significant at a *p* value of <0.05.

### 4.8. Experimental Design

The secretory protein changes in the RL95-2 cells, which occurred in response to trophoblastic cell-derived EVs (JAr cell EVs) vs. non trophoblastic cell-derived EVs (HEK 293 cell EV), were determined as follows:

The RL95-2 cells were seeded in 12-well plates and grown until they reached 85% confluency, as described above. After reaching the desired confluency, the cells were washed with Dulbecco’s phosphate-buffered saline without Ca^2+^ and Mg^2+^ (DPBS, Verviers, Belgium). The RL95-2 cells were then supplemented with EVs derived from trophoblast analog JAr cells at a concentration of 1 × 10^9^ nanoparticles/mL in EV-depleted medium. The nanoparticle concentrations were measured with a nanoparticle tracking analyzer, and similar concentrations of nanoparticle/mL from each EV source were used to treat the RL95-2 cells. As a control, the RL95-2 cells were treated with non-trophoblastic cell-derived EVs at similar concentration (HEK 293 cell EVs). Immediately after adding fresh media containing EVs to the cells, the cell culture supernatants were collected back into Eppendorf tubes and sequential centrifugation was performed. (0 h samples). Next, the RL95-2 cells were incubated with JAr and HEK cell-derived EVs for 24 h, and afterwards, the cell culture supernatants were collected (Figure S2). The proteomic profiles of the cell culture supernatants were analyzed using LC-MS/MS. The experiment was performed in triplicate on three different days. The protein fold change in the 0 h and 24 h samples was analyzed and used to identify proteins that were significantly changed in the RL95-2 cell secretome in response to JAr and HEK cell-derived EVs after 24 h. Proteins that uniquely changed in the JAr and HEK EV-treated RL95-2 cell secretome were sorted, and functional annotation and pathway overrepresentation analysis were performed. One differentially expressed protein marker, identified by mass spectrometry analysis, was validated using ELISA.

## 5. Conclusions

Trophoblast-derived EVs can change the endometrial cell secretory protein repertoire by harboring critical players of implantation, which is consistent with the findings of previous reports. These results suggest that embryonic signals can potentially regulate endometrial secretory protein responses, enhancing endometrial receptivity for the embryo implantation process. However, the exact mechanism by which embryonic EVs regulate these signaling pathways and their impact on structural and functional development, the attachment of the embryo, or the receptivity of the endometrium requires further study. Functional analyses of the effect of embryonic EVs on the endometrium may provide molecular insights into the process of embryo implantation, potentially identifying modulatory targets to treat implantation failure in humans and other mammalian species.

## Figures and Tables

**Table 1:** KEGG pathway enrichment analysis of differentially expressed proteins in JAr EV-treated RL95-2 cells.

KEGG Pathway	FDR	Fold Enrichment	Gene Names
Glutathione metabolism *	4.0 × 10^−1^	6.6	GSTP1, GSR, IDH1, PRDX6
Biosynthesis of amino acids *	4.40 × 10^−1^	6.6	GOT1, IDH1, MAT2A, PGK1
Metabolic pathways *	6.90 × 10^−1^	24.6	CNDP2, GPI, GOT1, GSTP1, GSR, HPRT1, ISYNA1, IDH1, LDHA, MIF, MAT2A, NIT2, PRDX6,PGK1, GALNT6
Carbon metabolism *	6.90 × 10^−1^	6.6	GPI, GOT1, IDH1, PGK1,
Cysteine and methionine metabolism *	7.40 × 10^−1^	4.9	GOT1, LDHA, MAT2A
Glycolysis/Gluconeogenesis *	9.4 × 10^−1^	4.9	GPI, LDHA, PGK1
Tight junction *	9.4 × 10^−1^	6.6	ACTB, MYH9, PCNA, PPP2R1A
Protein processing in endoplasmic reticulum *	9.4 × 10^−1^	6.6	RAD23B, CAPN1, HSP90AB1, LMAN2,
Peroxisome	9.7 × 10^−1^	4.9	IDH1, PRDX1, SOD1
Phenylalanine metabolism	1.0 × 10	3.3	GOT1, MIF
2-Oxocarboxylic acid metabolism	1.0 × 10	3.3	GOT1, IDH1

* Asterix indicates *p* ≤ 0.05.

**Table 2:** Differentially expressed proteins in the JAr EV-treated RL95-2 cell secretome with a potential role in the process of embryo implantation (evidence related to the embryo implantation process, including endometrial receptivity, embryo implantation, and early embryo development, is provided).

UniProt Accession	Gene Name	Protein Description	Protein Fold Change	Function	References
P05997	COL5A2	Collagen alpha-2(V) chain	5.28	A main protein component of the extracellular matrix and upregulated in receptive endometrium and implantation sites.	[32,33]
P12004	PCNA	Proliferating cell nuclear agent	4.32	Increased the expression of PCNA in stromal cells and myometrium with progressing gestation in rats and has a role in stromal cell proliferation.	[34]
P00390	GSR	Glutathione reductase	4.07	Reported as a potential human cumulus cell quality marker for pregnancy prediction. Known to regulate glutathione (a major antioxidant) production in cells; hence, it has a role in embryo development.	[35,36]
P31153	MAT2A	Methionine Adenosyltransferase 2A	3.91	Improves methionine-mediated DNA synthesis through the SAMTOR/mTORC1/S6K1/CAD pathway during human embryo implantation. Supports peri-conception embryo development in bovine.	[37,38]
O14980	XPO1	Exportin-1	3.86	Linked with repeated implantation failure by affecting the proliferation and differentiation of endometrial stromal cells in humans.	[39]
P17931	LGALS3	Galectin-3	3.95	Amplifies the inflammatory response; hence, it might play a role in early embryo implantation. Increased in the embryo implantation site and required for embryo implantation in mice. Increased LGALS3 induces endothelial cells morphogenesis and angiogenesis. Previously reported as being upregulated in Ishikawa cells treated with trophectoderm EVs (intracellularly).	[14,40,41,42,43]
P68036	UBE2L3	Ubiquitin-conjugating enzyme E2 L3	3.12	Only expressed in the uterus during pregnancy, supports embryo survival, and increases implantation potential in mice.	[44]
Q13753	LAMC2	Laminin subunit gamma-2	2.82	Interacting molecule in the embryo–endometrial interface.	[45]
P32119	PRDX2	Peroxiredoxin-2	2.82	Regulates trophoblast cell proliferation and apoptosis during early pregnancy and is mediated by c-Myc. Downregulation is linked with recurrent miscarriages. Previously reported as being upregulated in Ishikawa cells treated with trophectoderm EVs (intracellularly).	[14,27]
P50990	CCT8	T-complex protein 1 subunit theta	3.87	Altered in females with endometriosis during the window of implantation in humans.	[46]
Q08431	MFGE8	Lactadherin	2.75	Expressed in embryo–maternal interface in humans and equine. Known to play a role in embryo attachment to the endometrial epithelial cells. Increased secretion has been linked with stimulation by hCG. Previously identified as being significantly upregulated in Ishikawa cells treated with trophectoderm EVs (intracellularly).	[14,47,48,49,50,51]
P30041	PRDX6	Peroxiredoxin 6	2.78	Mediates antioxidant activity; hence, it is important for embryo development. Previously identified as changing intracellularly in Ishikawa cells treated with trophectoderm.	[14,52]
P00558	PGK1	Phosphoglycerate kinase 1	2.69	Increased in in vitro decidualization in endometrial stromal cells by regulating angiogenesis and glycolysis. Deficiency leads to impaired decidualization.	[53]
P52434	POLR2H	RNA polymerase II, I and III subunit H	2.66	Increased in endometrium in high fertility heifers in the midluteal phase of the estrous cycle.	[54]
Q8NCL4	GALNT6	Polypeptide *N*-acetylgalactosaminyltransferase 6	2.54	Upregulated in human blastocyst-stage embryos; potentially involved in the synthesis of oncofetal fibronectin, thus facilitating embryo attachment to endometrium.	[55]
P00338	LDHA	Lactate dehydrogenase A	2.35	Lactate dehydrogenase (LDH) isoform, LDHB (which favors pyruvate formation) is transformed to LDHA (which favors lactate formation) during the early phase of embryo implantation in blastocysts that can potentially support tissue invasion. Previously reported as being upregulated in Ishikawa cells treated with trophectoderm-derived EVs (intracellularly).	[14,31]
P31948	STIP1	Stress-inducible phosphoprotein 1	2.28	Lack of STIP1 causes embryonic lethality in mice.	[56]
P00441	SOD1	Superoxide dismutase 1	2.27	Activity peaks in the midluteal phase of the menstrual cycle in humans. Released by human embryos and found in IVF-spent media, but its relation to implantation potential is not clear. Has been linked with fertility capacity in mice. Previously reported as being upregulated in Ishikawa cells treated with trophectoderm-derived EVs (intracellularly).	[14,57,58,59]
P09211	GSTP1	Glutathione S-transferase Pi	2.11	Increased in the uterine fluid of pregnant mares compared to cyclin mares. Reduce inflammation by reducing cyclooxygenase-2 (COX-2). Previously reported as being upregulated in Ishikawa cells treated with trophectoderm-derived EVs (intracellularly).	[14,60]
P17987	TCP1	T-complex 1	2.1	Increased during pregnancy in horses and potentially play a role in the maternal recognition of pregnancy. Downregulated in pregnancy loss compared to healthy pregnancies. Necessary for folding newly synthesized proteins such as actin and tubulin. Previously reported as being upregulated in Ishikawa cells treated with trophectoderm-derived EVs (intracellularly).	[14,61]
P35579	MYH9	Myosin heavy chain 9	2.02	Loss of MYH9 is lethal to embryos and plays a key role in cytokinesis in mice.	[62]
P06744	GPI	Glucose-6-phosphate isomerase	2.53	Plays a role in glycolysis and is needed for embryo implantation in ferrets.	[63]
P14174	MIF	Macrophage migration inhibitory factor	2.43	A pro-inflammatory cytokine that showed a slight increase in the secretory phase of the menstrual cycle in humans.	[28,64]
P08238	HSP90AB1	Heat shock protein HSP 90-beta	2.18	Downregulated in human villi and decidua of early missed abortion patients. Play roles in placental development and cell proliferation in early mouse embryo development. Previously reported as being upregulated in Ishikawa cells treated with trophectoderm-derived EVs (intracellularly).	[14,65,66,67,68,69]

## Data Availability

The mass spectrometry proteomics data have been deposited to the ProteomeXchange Consortium via the PRIDE [1] partner repository with the dataset identifier PXD040311.

## Supplementary Materials

The following supporting information can be downloaded at: https://www.mdpi.com/article/10.3390/ijms241511924/s1.
