# Supplementary material for: Secretory Proteomic Responses of Endometrial Epithelial Cells to Trophoblast-Derived Extracellular Vesicles
Source: Int J Mol Sci. 2023 Jul 25;24(15):11924. doi: 10.3390/ijms241511924 (PMC10418763; doi:10.3390/ijms241511924)
Supplement: Supplementary file 1 [file ijms-24-11924-s001.zip › ijms-2490612-supplementary.pdf]

Supplementary Figure S1

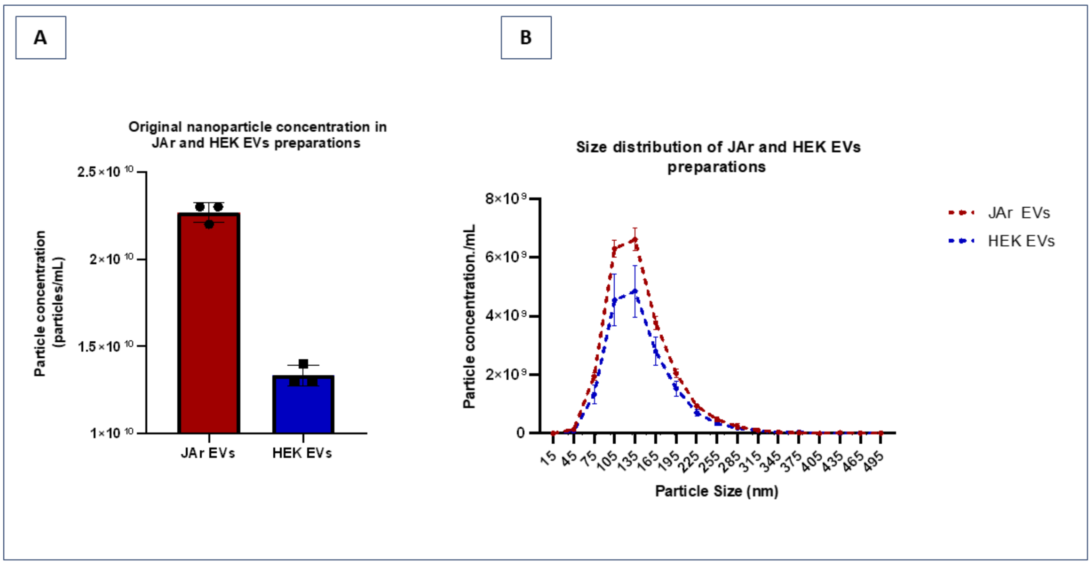

Figure S1. Nanoparticle concentration and size profiles of JAr and HEK derived EVs A: Nano particle concentration in JAr and HEK 293 cell derived EVs preparations. B: Size distribution of JAr and HEK cell derived EVs preparations

Supplementary Figure S2

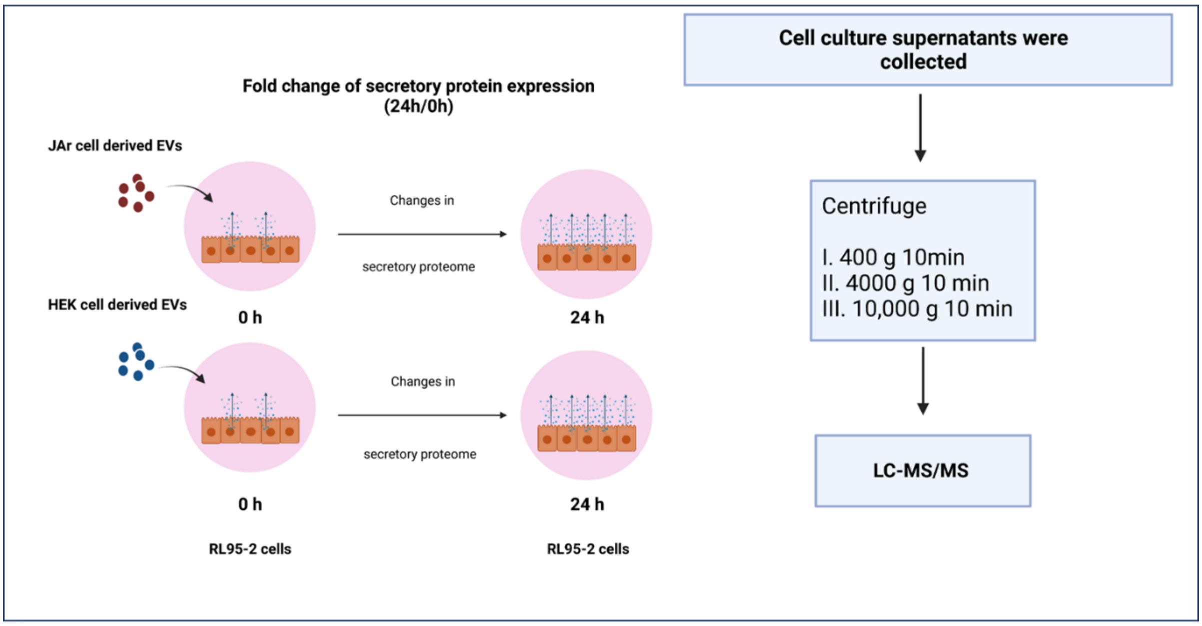

Figure S2. Experimental design for determining the secretory proteomic alterations of RL 95-2 cells in response to trophoblast cell derived EVs (JAr EVs) vs. non-trophoblast cell EVs (HEK EVs).

Cell culture supernatants were collected at 0 h and 24 h of EV treatment and processed to remove contaminating cells and debris. Secretory proteomic profiles of cell culture supernatants were analysed by LC-MS/MS, and fold changes of protein expression between 0 h and 24 h samples were calculated for JAr and HEK EVs treated groups separately and compared.

Supplementary Figure S3

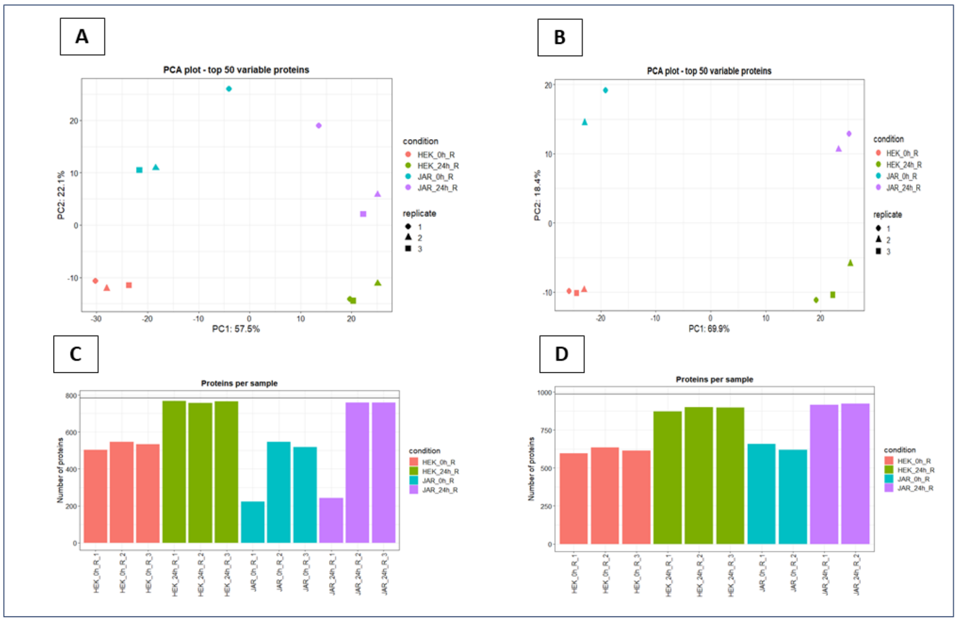

Figure S3. Data pertaining to the detection of outliers observed in proteomic data analysis. A and B. Principal Component Analysis of JAr and HEK EVs treated RL95-2 cell groups. C and D. Total number of proteins identified in each individual sample before and after removing the outlier. JAr EVs treated RL95-2 secretory protein profile at 0 h; JAR_0h_R, JAr EVs treated RL 95-2 secretory protein profile at 24 h; JAR_24h_R, HEK EVs treated RL 95-2 secretory protein profile at 0 h; HEK_0h_R, HEK EVs treated RL 95-2 secretory protein profile 24 h; HEK_24 h_R. Here, one replicate of JAr EV treated RL95-2 cell secretory protein profile (oh and 24 h) was observed as an outlier (due to batch effect) and removing it greatly improved the clustering of the data as shown in figure B.

Supplementary Figure S4.

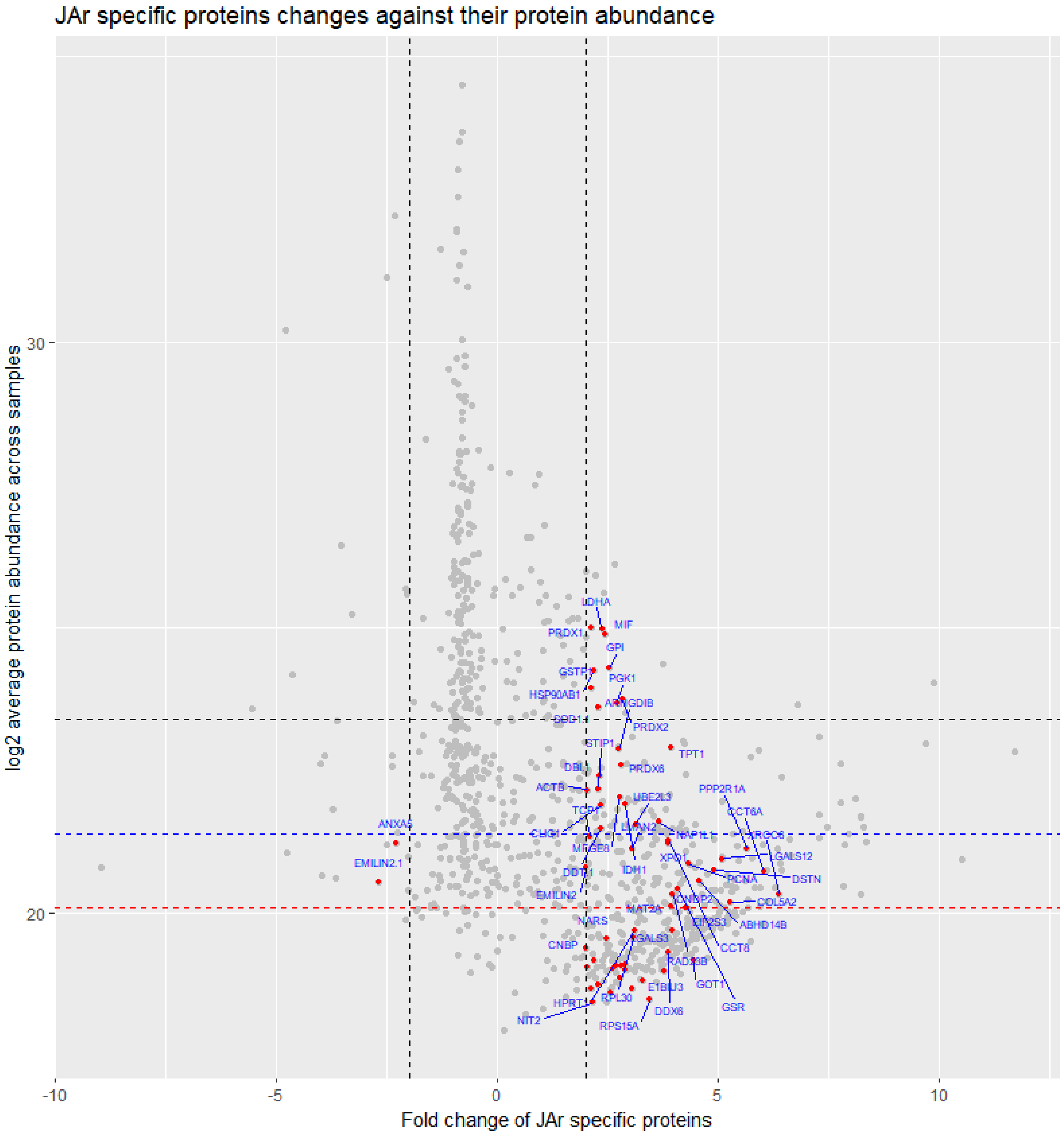

Figure S4. Distributions of protein abundance of proteins specifically altered in JAr EVs treated RL95-2 cell secretome.

(A) The fold change values of proteins specifically altered in JAr EV treated RL95-2 cell secretome from 0 h to 24 h was plotted against distribution of log transformed and normalized average protein abundance across samples, with the first quartile (Q1 in red), median (in blue), and third quartile (Q3 in black) indicated by horizontal bars. More than half the identified proteins (38/64) proteins falls above Q1 (above the relatively lowest abundant protein group) with 9 proteins in the highest abundant protein group (above Q3).

Supplementary Figure S5.

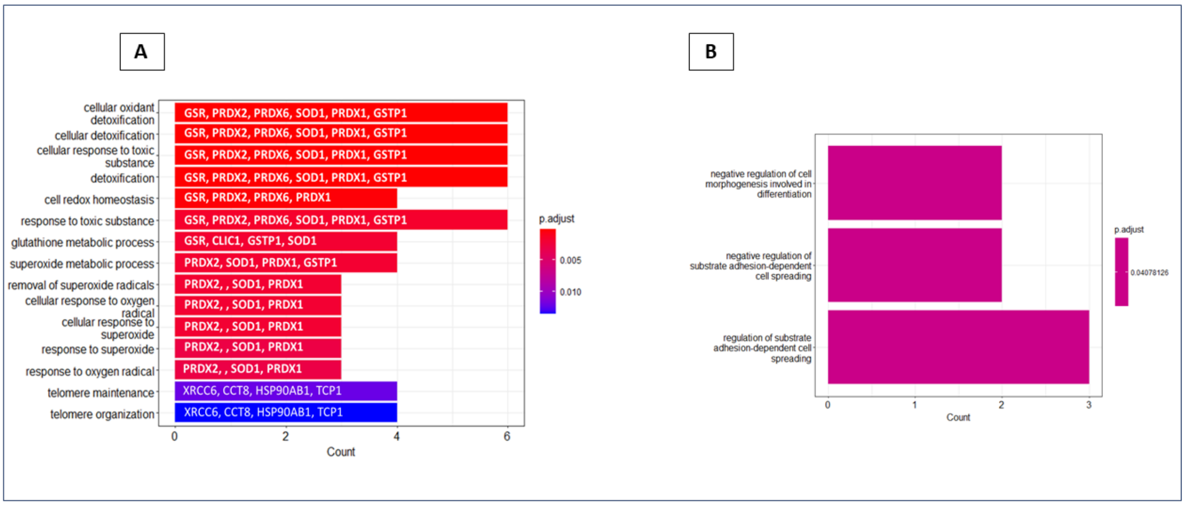

Figure S5. Functional annotation and GO enrichment pathway analysis **A.** Functional annotation and GO enrichment pathway analysis of proteins altered uniquely in the JAr EV treated group from 0 to 24 h. **B**. Functional annotation and GO enrichment pathway analysis of proteins uniquely altered in the HEK EV treated group from 0 h to 24 h. p.adju < 0.05 considered statistically significant.

Table S1. KEGG pathway analysis of proteins specifically identified in RL95-2 cell secretome as changing in HEK EVs treated group from 0 h to 24 h are shown below.

**KEGG pathway**	***p* value**	**FDR**	**Fold enrichment**	**Gene names**
Proteasome	3.6×10^{11}	3.8×10^{9}	13.6	PSMA5, PSMA6, PSMB1, PSMB2, PSMB4, PSMB5,PSMB6,PSMB7, PSME2
Spinocerebellar ataxia	5.0×10^{6}	2.6×10^{4}	12.1	PSMA5, PSMA6, PSMB1, PSMB2, PSMB4, PSMB5,PSMB6,PSMB7, PSME2
Huntington disease	9.6×10^{5}	3.4×10^{3}	13.6	CLTC, PSMA5, PSMA6, PSMB1, PSMB2, PSMB4, PSMB5,PSMB6,PSMB7
Parkinson disease	2.7×10^{4}	6.6×10^{3}	12.1	PSMA5, PSMA6, PSMB4, PSMB5,PSMB6,PSMB7
Prion disease	3.1×10^{4}	6.6×10^{3}	12.1	PSMA5, PSMA6, PSMB1, PSMB2, PSMB4, PSMB5,PSMB6,PSMB7
Amyotrophic lateral sclerosis	1.7×10^{3}	3.0×10^{2}	12.1	PSMA5, PSMA6, PSMB1, PSMB2, PSMB4, PSMB5,PSMB6,PSMB7
Alzheimer disease	2.3×10^{3}	3.5×10^{2}	12.1	PSMA5, PSMA6, PSMB1, PSMB2, PSMB4, PSMB5,PSMB6,PSMB7
Bacterial invasion of epithelial cells	6.2×10^{3}	8.1×10^{2}	6.1	ARPC1B, ARPC3, CDH1, CLTC
Pathways of neurodegeneration - multiple diseases	7.6×10^{3}	8.9×10^{2}	12.1	PSMA5, PSMA6, PSMB1, PSMB2, PSMB4, PSMB5,PSMB6,PSMB7
Shigellosis	3.2×10^{2}	3.3×10	7.6	CD14, SKP1, ARPC1B, ARPC3,ACTN1
Pathways in cancer	4.3×10^{2}	4.1×10	10.6	SKP1, CDH1, CSF2RA, ELOB, GST01, JUP, LAMB1
Tight junction	4.9×10^{2}	4.1×10	6.1	ARPC1B, ARPC3,ACTN1, MYL6
Protein processing in endoplasmic reticulum	5.1×10^{2}	4.1×10	6.1	SKP1, SIL1, P4HB, PDIA6
Amoebiasis	8.9×10^{2}	6.7×10	4.5	CD14, ACTN1, LAMB1,
